# Comparison of AFM Nanoindentation and Gold Nanoparticle Embedding Techniques for Measuring the Properties of Polymer Thin Films

**DOI:** 10.3390/polym11040617

**Published:** 2019-04-03

**Authors:** Guojun Jiang, Sheng Xie

**Affiliations:** 1 Department of Science, Zhijiang College of Zhejiang University of Technology, No. 958 Yuezhou Road, Shaoxing 312000, China; jiangguojun1986@126.com; 2 College of Material and Textile Engineering, Jiaxing University, No. 118 Jiahang Road, Jiaxing 314000, China

**Keywords:** AFM nanoindentation, gold nanoparticle embedding, properties of thin film

## Abstract

The surfaces of polymer and interfaces between polymer and inorganic particles are of particular importance for the properties of polymers and composites. However, the determination of the properties of surfaces and interfaces poses many challenges due to their extremely small dimensions. Herein, polystyrene and polymethyl methacrylate thin film on silicon wafer was used as a model system for the measurement of the properties of the polymer near free surface and at the polymer-solid interface. Two different methods, i.e., nanoindentation using atomic force microscopy (AFM) and the gold nanoparticle embedding technique, were used for these measurements. The results showed the elastic modulus of PS near the free surface determined by nanoindentation was lower than the bulk value. Based on contact mechanics analysis, nanoparticle embedding also revealed the existence of a lower-modulus, non-glassy layer near the free surface at temperatures below the bulk glass transition temperature (*T*_g_). However, near the polymer-solid interface, the AFM nanoindentation method is not applicable due to the geometry confinement effect. On the other hand, the nanoparticle embedding technique can still correctly reflect the interactions between the polymer and the substrate when compared to the ellipsometry results.

## 1. Introduction

Properties near polymer-air surface and polymer–solid interface play a critical role in many technological applications such as thin films, coating, adhesives and polymer-based micro/nanoscale devices and constructers [1,2]. It has been demonstrated that the molecular structure and thermodynamics near the interface could be different from those of bulk, leading to a significant deviation in glass transition temperature (*T*_g_) and modulus [3,4]. These differences are important for the polymer nanocomposites since the properties of the interfaces between the nanoparticle and polymer matrix play a critical role in dictating the properties of the nanocomposites. Both nanoparticle surface and polymer matrix characteristics show significant effect on the properties of the interfaces. These characteristics induced different interactions, and usually, strong interactions; for example, hydrogen bond, ionic bond, and covalent bond, lead to high interface properties, while weak interactions induced low interface properties [5,6]. For example, a strong interaction between poly(methyl methacrylate) (PMMA) or poly(2-vinyl pyridine) (P2VP) with silica nanoparticles due to hydrogen bonding increases the *T*_g_ of these two polymer nanocomposites [7,8,9]. On the other hand, a *T*_g_ decrease was reported when the same silica nanoparticle was added into PS due to poor interfacial interactions between the PS and the silica caused by the non-wetted interfaces [10]. Therefore, it is extremely important to have a fundamental understanding on how thermal and mechanical properties of polymer vary near the vicinity of the free surface and the polymer-solid interface. However, the randomly dispersed nanoparticles and the extremely small size of the interfacial region make it very difficult to directly investigate the interfacial interactions in nanocomposites. In recent years, several researchers have used supported polymer thin films in which polymer film and substrate are used to mimic polymer matrix and nanoparticle, respectively, to investigate the interface in nanocomposites. The well-defined large interfacial area in the supported thin film system makes it an excellent one-dimension model for measuring polymer-solid interfacial interactions. Studies on supported polymer thin films have indicated that the *T*_g_ values of the polymer films are strongly influenced by substrate properties [7,8]. Bansal et al. [10] correlated the thin film *T*_g_ with the bulk *T*_g_ of a model PS-silica nanocomposite by drawing a direct analogy between the film thickness and the interparticle spacing in nanocomposites. Torkelson et al. [11] proposed a model nanocomposite using polymer thin films confined between two slides. *T*_g_ and physical ageing in the confined polymers were measured by a fluorescence method.

To explore the thin film properties, many analytical techniques such as relaxation of fluorescence [12], ellipsometry [13], and positron annihilation lifetime spectroscopy [14] have been developed. However, most of them are limited to the measurement of *T*_g_ or free volume of polymers. For measuring mechanical properties of thin films, some technologies have also been developed. When a thin polymer film is placed on a thick and soft substrate, the shrinkage of the pre-stretched soft substrate induces buckling of the thin polymer film, and the spacing of these highly periodic wrinkles highly depends on the elastic modulus of the film. Using this method, elastic modulus of many polymer films with different thicknesses were measured [15,16]. However, this method is just applicable for free-standing thin film, and the modulus of the whole film, instead of local modulus, is measured. Nanoindentation method using atomic force microscopy (AFM) or scanning force microscopy (SFM) is a traditional method for the measurement of material surface. By control the penetration depth, the modulus of the surface layer with specific thickness, i.e., local modulus, can be measured. Applying a precise control of the penetration depth at the nanometer scale and a normal load in the range of nanonewtons to piconewtons, the depth profile of elastic modulus of PS films from free surface to bulk was obtained by Miyake et al. [17]. However, their measurements were far away from the polymer-solid interfacial region [18].

Nanoparticle embedding technique has been used to measure the surface layer near the polymer free surface at temperatures below the bulk *T*_g_ [19]. In contrast to the nanoindentation method in which the penetration is a dynamic process, the nanoparticle embedment can be performed in a much longer time, e.g., several hours, to achieve full relaxation of polymer chains.

In this study, PMMA and PS thin films supported on silicon wafer were used to investigate the interactions of these two polymers with silica surface. *T*_g_s of the films with different thicknesses were measured by ellipsometry, from which a strong interaction between PMMA and silica surface, while a weak interaction between PS and silica surface was demonstrated. Subsequently, the surface properties were investigated by gold nanoparticle embedding and AFM nanoindentation. It was shown that both methods could be used for the measurement of surface modulus, which is lower than that of bulk; whereas for the interface layer that existed in a very thin film, the results of nanoindentation was contradictory to that from the ellipsotmetry measurement, but the nanoparticle embedding results correctly reflected the interactions between polymer films and substrate. This work not only demonstrated the limitation of nanoindentation in the study of interface properties, but also extended the gold nanoparticle embedding method to the measurement of surface/interface modulus, making it capable to investigate the property profile of polymer thin films from the free surface to the polymer-substrate interface.

## 2. Experimental

### 2.1. Preparation of Supported Thin Films

Solutions of PS (*M*_n_ = 214,000, *M*_w_/*M*_n_ = 1.03) and PMMA (*M*_n_ = 212,900, *M*_w_/*M*_n_ = 1.06) from Polymer Source Inc. (Montreal, Quebec, Canada) were prepared by dissolving the polymer in toluene. They were then filtered through an inorganic membrane with a pore size of 0.1 μm (Whatman, Anotop 25, UK). The supported polymer thin films were prepared by spin-coating the solution onto a fresh-cleaned silicon wafer. The film thickness was controlled by the solution concentration and the spinning speed. Films were dried at ambient temperature for 48 h and then annealed at 135 °C for 12 h under vacuum to remove the remaining solvent and relieve any residual stresses built in the film during spin coating. After annealing, films were slowly cooled to room temperature under vacuum. The thickness of the films was determined using Nanospec 1000 spectroscopic reflectometry ( Nanometric Co., Milpitas, CA, USA) and the nanometrice roughness of the film was characterized by Nanoscope II AFM ( Veeco Instruments Inc., Plainview, NY, USA) and confirmed to be less than 1 nm.

### 2.2. Ellipsometry

The average *T*_g_s of thin films were measured by a Uvisel spectroscopic phase modulated ellipsometer (Horiba Jobin Yvon, Edison New Jersey, NJ, USA) with a wavelength range from 400 to 830 nm and an incident angle of 70°. The films’ thicknesses were determined by fitting the Ψ and Δ spectra. A hot stage with accuracy of ±0.1 °C was used to control the temperature during *T*_g_ measurements with a heating rate and equilibration time of 1 °C/min and 15 min, respectively. The thickness expansions in the glassy and rubbery regions were linearly fitted with the least square. The glass transition temperature was defined as the temperature at which the linear constructions intersect.

### 2.3. AFM Nanoindentation

The nanoindentation was carried out using a Nanoscope IIIA Multimode AFM (Veeco Instruments Inc., Plainview, NY, USA) in air at room temperature. The spring constant and indenter radii (determined by Scanning Electronic Microscopy, SEM, S-4800, Hitachi, Tokyo, Japan) of silicon cantilevers used in this study are about 20 N/m and 50 nm, respectively. The indenter before and after indentation measurement was characterized by SEM to make sure that no damage was caused during the experiment.

The force-penetration depth curves for the modulus calculation were obtained by the perpendicular motion of the cantilever until reaching the pre-specified penetration depth. During the indentation, the z direction displacement of the cantilever was controlled by a piezoelectric actuator. The force produced during penetration was calculated through the recorded vertical deflection and the spring constant of the cantilever. The actual penetration depth is defined as

(1) $$\delta =\left(z-z_{0}\right)-\left(d-d_{0}\right)=\left(z-d\right)-\left(z_{0}-d_{0}\right)$$

where the reference point ($z_{0}$,$d_{0}$) is the point of zero indentation, *z* is the piezoelectric actuator motion distance, and *d* is the vertical deflection of the cantilever.

### 2.4. Gold Nanoparticle Embedding

Unconjugated gold colloid with a particle size of 20.4 ± 8% nm was supplied by Ted Pella, Inc (Redding, CA, USA). The colloid was diluted 50-fold with 18 MΩ·cm water before use. A drop of diluted gold colloid was placed on top of each film and then allowed to dry at room temperature. Samples were annealed at pre-specified temperatures under vacuum for overnight. After reaching the equilibrium state, the system was quenched with ice and then taken out for the AFM tapping mode measurement. The average height of the gold nanoparticles on bare silicon surface was first measured, and then the heights of the gold nanoparticles on polymer films under different conditions were determined. The embedding depth of the gold nanoparticle was the difference between the height on bare silicon wafer and polymer thin film.

## 3. Results and Discussion

**Figure 1:**
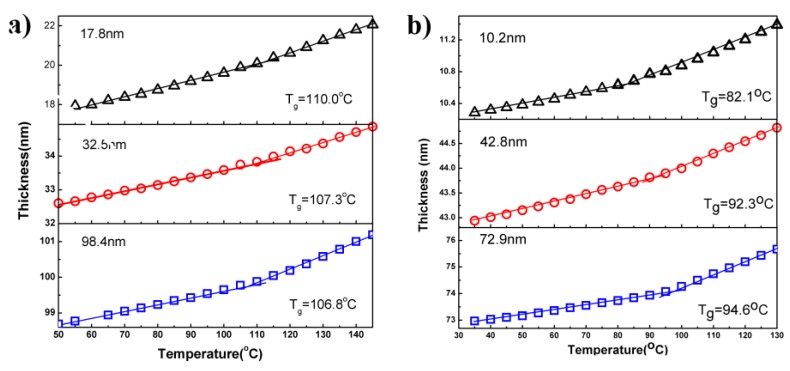
*T*_g_s of (**a**) poly(methyl methacrylate) (PMMA) and (**b**) PS thin films on the Si substrate determined by ellipsometery.

To investigate the interactions between different polymers with silica surface, the *T*_g_s of PMMA and PS thin film with different thicknesses on silicon substrate were measured by ellipsometry, as shown Figure 1a,b. It is clear that the *T*_g_s of these two films highly depended on the film thickness, but the trend was completely opposite. The *T*_g_s of PMMA thin films increased slightly as decreasing film thickness. The *T*_g_ values for the PMMA thin films of 98, 32, and 18 nm of are 106.8, 107.3, and 110 °C, respectively. These results agree well with other studies, indicating that PMMA has a strong interaction with the silica surface. On contrary, the *T*_g_ values of the PS film decreases when the thickness was reduced. For the PS film of 73 nm, *T*_g_ was 94.6 °C, whereas when the thickness was decreased to around 11 nm, *T*_g_ was decreased to 82.1 °C. The decreased *T*_g_ of PS thin film on silica surface is likely due to the weak interaction between PS and silica surface due to their opposite characteristics, i.e., hydrophobicity and hydrophilicity, respectively.

**Figure 2:**
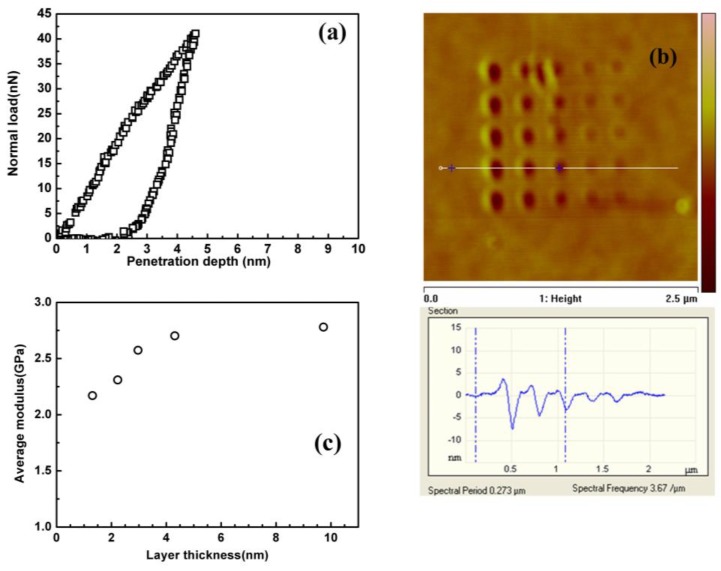
(**a**) Typical force-penetration depth, (**b**) AFM image, and (**c**) the variation of elastic modulus versus the indentation depth for PS thin film obtained by nanoindentaion.

The surface properties of polymer films was investigated by nanoindentation. Herein, PS film with a thickness of 120 nm was used. From literature, this film is thick enough to avoid the effect of substrate on the surface. During the penetration of the AFM tip into the polymer films, the cantilever was deformed, from which the force exerted on the tip could be calculated. Therefore, a force-penetration depth curve could be obtained by simultaneously recording the deformation of cantilever and penetration depth, as shown in Figure 2a. To investigate the change of the properties of PS from surface to the bulk along the thickness direction, the tip was penetrated into the film with different depths by adjusting the force loaded on the indenter. To confirm the accuracy of the results, the tip before and after the nanoindentation experiment was observed using SEM (Figure S1). It can be seen that there was no damage of the tip during this experiment. Figure 2b shows the AFM image of the PS film penetrated by AFM tip under a different force. Obviously, the depth of the residual hole produced by nanoindentation increased as the force load increased. Using the force-penetration curves, the average elastic modulus of layers with different thicknesses beneath the free surface can be estimated by using the Sneddon’s formula (2).

(2) $$E_{r}=\frac{\sqrt{\pi }S}{2\beta \sqrt{A_{proj}}}$$

where $\;E_{r}=\{[(1-v_{1}^{2})/E_{1}]+[(1-v_{2}^{2})/E_{2}]{\}}^{-1}$ is the composite elastic modulus; $E_{1}$, $E_{2}$, $v_{1}$, and $v_{2}$ are elastic modulus and Poisson’s ratio of the film and indenter, respectively. *S* is the slope of the unloading curve at the maximum load, *A_proj_* is the projected area tip-shape contact, *β* is the tip shape parameter which is equal to 1.0 in this case because of the circular contact. Figure 2c shows the calculated average elastic modulus as a function of indentation depth. It can be observed that the average elastic modulus near the film surface (3~4 nm) is lower than the bulk modulus. The elastic modulus increases with the penetration depth and reaches the bulk value (2.8 GPa) when the depth is around 10 nm. This result is similar to that reported by Miyake et al. [17].

**Figure 3:**
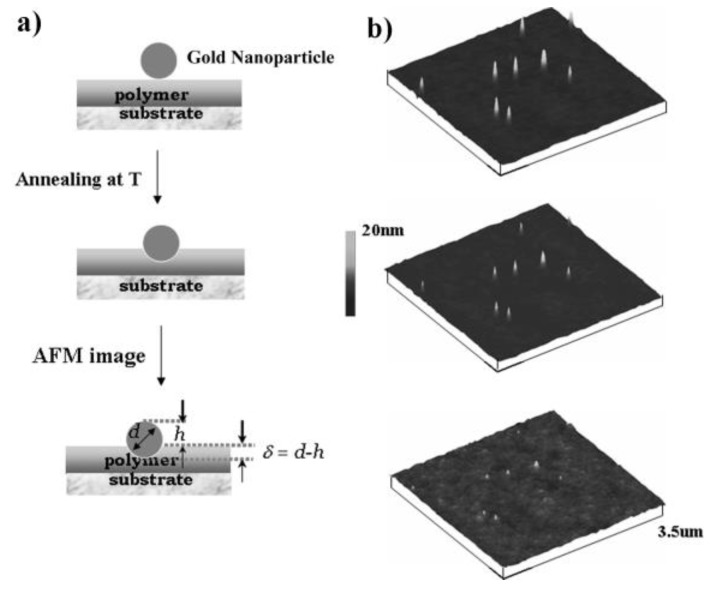
(**a**) Schematics of nanoparticle embedding technique. (**b**) AFM images of nanoparticle on 120 nm PS film annealed at 90 °C (**Top**), 100 °C (**Middle**) and 110 °C (**Bottom**) in vacuum.

**Figure 4:**
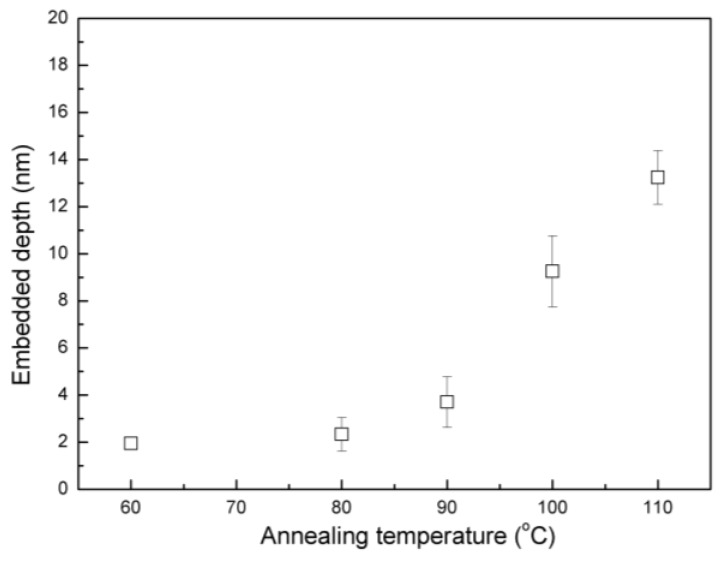
Embedded depth of 20 nm gold nanoparticle into 120 nm PS film in vacuum at various annealing temperature.

In parallel, gold nanoparticle embedding method was also used to investigate the surface properties of the PS film with a thickness of 120 nm. For this experiment, gold nanoparticles with a particle size of 20 nm were used. TEM and particle size statistic results in Figure S2 indicate the particle size and uniform distribution. For the AFM measurement, Teichroeb et al. [20] compared two different methods-one is scanning different spots on the sample to get an image of a fairly large number (~40) of particles for statistics, and the other is using a kinematics mounting hot stage to allow scanning the same spot at each time. The latter can acquire the same final precision without the necessity for imaging a large number of particles. In this study, we made a mark on the sample surface to make sure imaging the same spot each time, allowing us to track at least ten particles embedding with a fraction of nm precision. The schematic illustration and typical AFM images are shown in Figure 3a,b, respectively. We can see that with the increased annealing temperature, the gold nanoparticle (particles are same in three images) gradually embedded into the polymer films. The embedding depth was calculated as shown in schematic illustration, where *d* is the diameter of the gold nanoparticle on bare silicon wafer determined by AFM, and the *h* is the height of the gold nanoparticle after the embedding. To investigate the surface properties of PS film, the gold nanoparticles were placed on PS film surface, and a series of annealing temperatures were applied to investigate the dependence of embedded depth on temperature. Figure 4 depicts the embedded depths of gold nanoparticles with 20 nm diameters under vacuum at various annealing temperatures. The embedment of nanoparticles was observed at temperatures below bulk *T*_g_ of PS and the embedded depth increased with increasing the annealing temperature. When the annealing temperature was at 90 °C, the embedded depth of gold nanoparticles was around 3.7 nm, which agreed well with the value reported in literature and this value can be attributed to the thickness of the surface layer with a lower modulus [21]. Although the nanoindentation method provides a modulus profile in the PS film while the result of gold nanoparticle embedding displays a lower-modulus surface layer, they show a similar trend qualitatively. Namely, the mechanical strength of PS near the free surface is lower than that in bulk.

**Figure 5:**
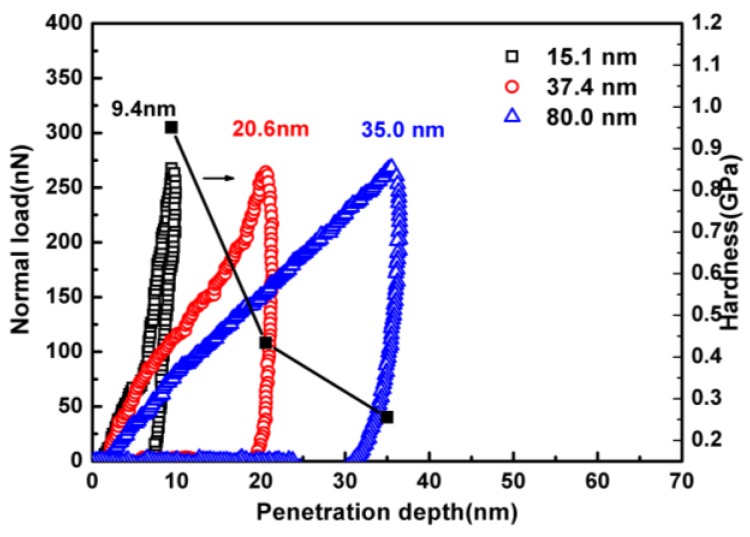
Force-penetration depth curve and hardness of PS thin film with different thickness on Si substrate obtained by nanoindentation.

In order to investigate the interfacial interactions between the polymer and the substrate, nanoindentation was conducted on PS films with reduced thickness. Figure 5 shows the force-penetration depth for the PS thin films with different thicknesses. The difference, in particular, the penetration depth among the curves can be clearly observed. When the same normal load was exerted on the indenter, the penetration depth in the thinner film (11.3 nm) is much smaller than that into the thicker film (80 nm). The hardness of these films calculated from Equation (3) is also shown in Figure 5.

(3) $$H=P_{\max}/A_{c}$$

where *P*_max_ is the maximum load during the nanoindentation and *A*_c_ is the contact area between the indenter and the thin film. The results imply that thinner PS film coated on the silicone substrate has a higher hardness. The same trend was also observed for the PMMA thin films.

**Figure 6:**
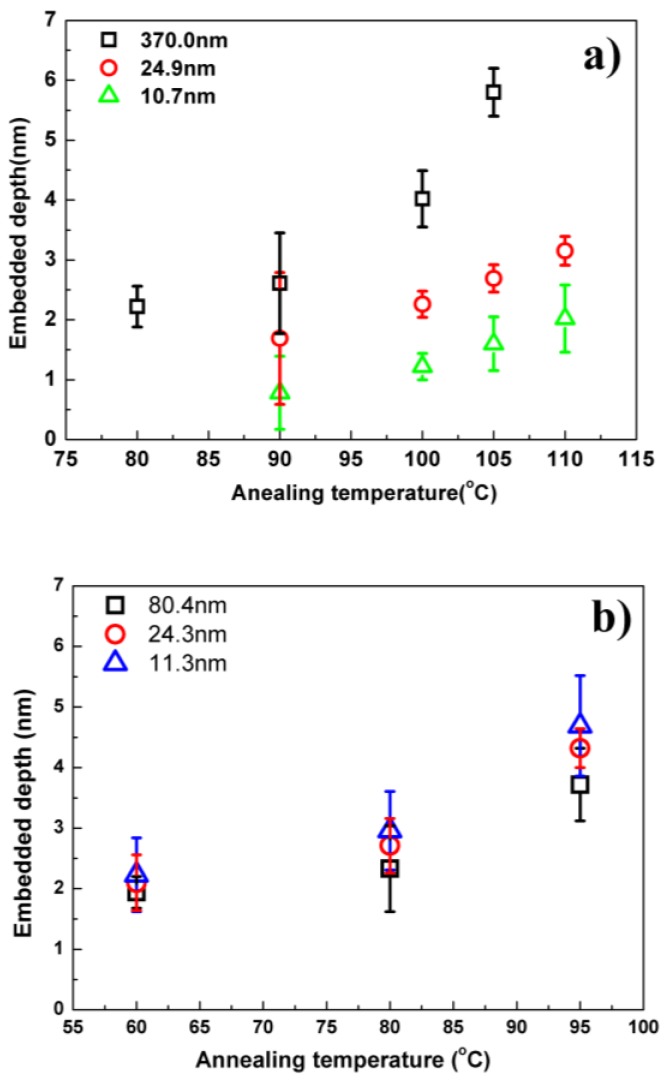
Embedding depth of gold nanoparticle versus annealing temperature on (**a**) PMMA and (**b**) PS thin film with different thicknesses on silicon substrate.

Figure 6a,b depicts the embedded depths of nanoparticles on the PMMA and PS films with different thicknesses as a function of the annealing temperature. For PMMA films, it can be observed that the embedded depth shifted to a low level with the reduced film thickness, indicating a strong polymer–substrate interaction, which makes it more difficult for nanoparticles to embed into the thinner films. For example, the gold nanoparticles can embed around 3.7 nm at 100 °C for the 370 nm film. Once the thickness is decreased to about 10 nm, the embedded depth becomes much lower, e.g., 1.3 nm. These results clearly suggest a strong chain confinement of PMMA near the SiO_2_ surface. It is well known that PMMA can form hydrogen bonding with silicon wafer containing hydroxyl groups. These attractive interactions reduce the segmental mobility and hence increase the *T*_g_ and modulus in the PMMA-silica nanocomposites [11] and PMMA films coated on the Si substrate.

A different embedding behavior is observed for the PS thin films as shown in Figure 6b. At the same annealing temperature, the values of gold nanoparticle embedding depth into the thinner films are higher than those into the thicker films. For the 80 nm film, the embedded depth was around 3.8 nm when the annealing temperature was at 95 °C. When the film thickness decreased to 24 nm and 11 nm, the embedding depths became 4.3 and 4.7 nm, respectively. The poor wetting, and hence poor bonding, between PS and silica nanoparticles has been reported in literature. Bansal et al. [10] observed the cavities around silica nanoparticles in PS/silica nanocomposites using scanning electronic microscopy (SEM) and attributed the *T*_g_ reduction of nanocomposites to the presence of free surface at the non-wetted interface. When PS was spin-coated on the silicon wafer with native oxide surface, the arrangement of chain molecules in the interfacial region were affected by this de-wetting interaction. Positron annihilation lifetime (PAL) study [14] also showed a significant density depression (0.4 g/cc) in the interface region between PS and Si substrate. The reduced density, meaning increased free volume and chain mobility, suppresses the *T*_g_ values of thin films and makes it easier for the gold nanoparticles to embed in.

**Figure 7:**
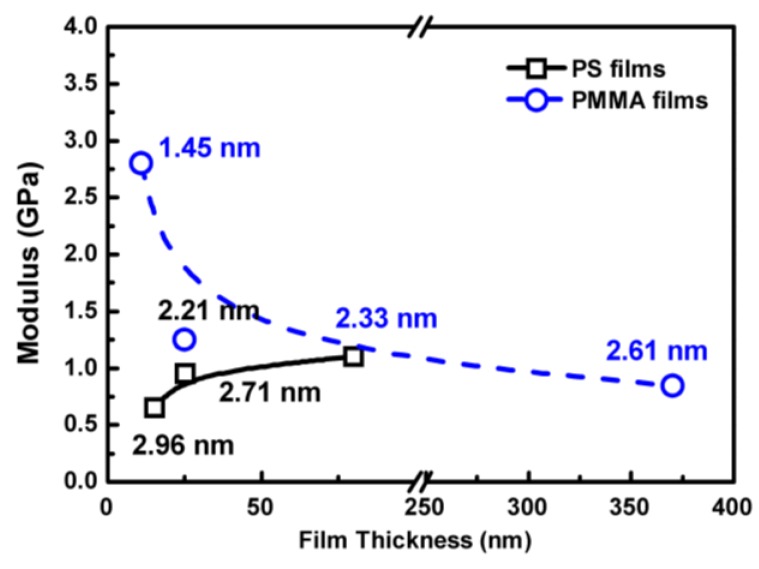
Calculated surface modulus of PS (at 80 °C) and PMMA (at 90 °C) films with different thickness. The number is the thickness of surface layer with the average modulus.

To interpret the embedding of the gold nanoparticle, Maugis model [22], as shown in Equation (4), is applied here to associate the embedded depth with the modulus of the polymer films.

(4) $$\delta =\frac{a}{2}\ln\frac{R+a}{R-a}-\sqrt{\frac{8\pi aw_{A}}{3K}}$$

where $K=3/4\left[\frac{E}{1-v^{2}}\right]$ is the equivalent elastic constant, *P* is the external loading on gold nanoparticle which is zero in this study, *E* and *v* are the elastic modulus and Poisson’s ratio of elastic material, *R* is the radius of the rigid nanoparticle, and *a* is the contact radius which has the relationship with *δ* as $\alpha ={\left[R^{2}-{\left(R-\delta \right)}^{2}\right]}^{1/2}$. The work of adhesion $w_{A}=\gamma_{1}+\gamma_{2}-\gamma_{12}$ is related to the surface energies of the two materials, $\gamma_{1}$ and $\gamma_{2}$, and their interfacial energy $\gamma_{12}$. Herein, the embedding depths were measured, adding the material constants of PS, PMMA, and gold nanoparticle [23,24], and the average modulus of the surface layer with the thickness of the imbedded depth can be calculated from Equation (4). For example, the gold nanoparticle can embed 2.33 nm into the 120 nm PS film. According to the Maugis model, the calculated modulus is 1.1 GPa. It means that the surface layer with a thickness of 2.33 nm has the average modulus of 1.1 GPa. The calculated surface modulus of PS and PMMA films with different thicknesses are shown in Figure 7. It is clear that with the decreased thickness, the surface modulus of PS and PMMA films decreases and increases, respectively, with the decreasing thickness due to the different interactions between the polymer and the substrate.

**Figure 8:**
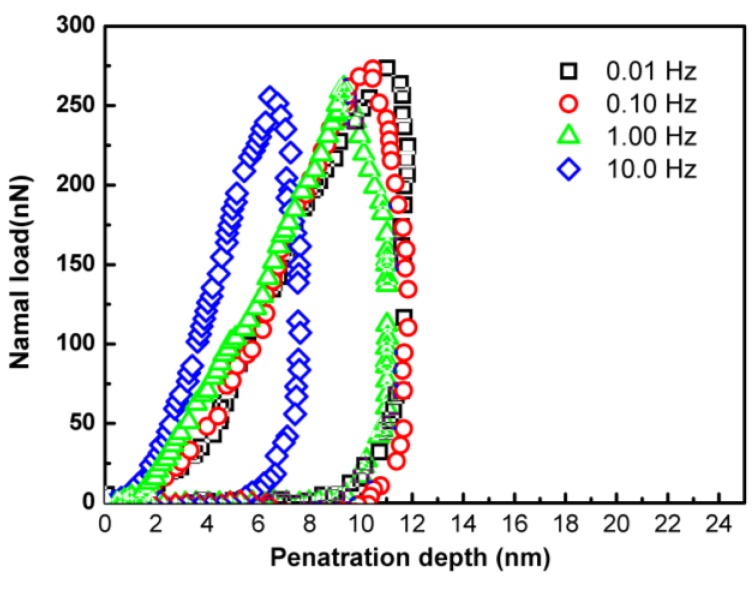
The force-penetration depth curves of nanoindentation at different penetration rate, the film thickness is 12.5 nm.

Associating the nanoindentation and nanoparticle embedding results with the *T*_g_ change of the interfacial region of the PS thin films on silicon wafer determined by ellipsometry, the nanoparticle embedding technique showed a correct trend while the AFM nanoindention technique provided a wrong trend. This is due to the geometry confinement effect of the substrate. For nanoindentation, both experimental and finite element analysis indicate that the polymer confinement between the indenter and the substrate can produce a significant influence on the mechanical behavior due to the structural changes associated with the constrained chain movement between the indenter/substrate gaps [25]. When the substrate has a higher modulus, the calculated modulus of thin films from the force-penetration depth curve tends to be higher due to the confinement effect of the substrate stiffness. With the decreased film thickness, the spatial confinement effect introduced by the indenter and the substrate on the nanoindentation measurement would become more severe. For nanoindentation performed by AFM, the whole loading-unloading process is completed in minutes, i.e., the penetration rate is several nanometers per second. The embedment of nanoparticles, however, takes much longer time, around one hour embedding time to reach equilibrium for 20-nm gold spheres [19]. This means that the penetration rate is in the order of several nanometers per hour. This rate difference may explain the different results obtained by the two methods. In the case of nanoparticle embedding, the very low penetration rate allows the polymer chains between the indenter (i.e., gold nanoparticles) and the substrate to relax, leading to a much reduced geometry confinement effect. To test this hypothesis, the nanoindentaion with different penetration rates was carried out and the results are presented in Figure 8. It can be seen that the penetration depth and hardness monotonously increase and decrease, respectively, with the decreasing penetration rate at the same load. This observation agrees with the simulation results of nanoindentation of viscoelastic materials that an increased load is needed to obtain the same penetration depth when penetration rate increases [26]. However, the nanoindentation results still show a remarkable deviation from the actual even at the lowest penetration rate (0.01 Hz).

## 4. Conclusions

In summary, two different methods, i.e., nanoindentation and gold nanoparticle embedding methods, were used to investigate the surface and interface properties of supported polymer thin films. Specifically, two polymers, PS and PMMA which have different interactions with silica surface were used for the interface property study. The results showed that for the surface property measurement from the relatively thick films where the effect of the substrate on surface could be negligible, both methods showed reasonable results that the modulus near the polymer surface was lower than that of bulk. However, for interface property study using very thin polymer film, the weak interaction between PS and silica surface could not be investigated by the nanoindentation method due to the geometry confinement effect near the substrate surface. On the other hand, the gold nanoparticle embedding technique can show a correct trend of the interfacial interactions between the polymer and the substrate.

## Supplementary Materials

The supplementary materials are available online at https://www.mdpi.com/2073-4360/11/4/617/s1.
