# Supplementary material for: Comparison of AFM Nanoindentation and Gold Nanoparticle Embedding Techniques for Measuring the Properties of Polymer Thin Films
Source: Polymers (Basel). 2019 Apr 3;11(4):617. doi: 10.3390/polym11040617 (PMC6523445; doi:10.3390/polym11040617)
Supplement: Supplementary file 1 [file polymers-11-00617-s001.pdf]

Supporting Information

Comparison of AFM Nanoindentation and Gold Nanoparticle Embedding Techniques for Measuring the Properties of Polymer Thin Films

Guojun Jiang[1], Sheng Xie[2]*

[1]Department of Science, Zhijiang College of Zhejiang University of Technology, No.958 Yuezhou Road, Shaoxing, 312000, P.R. China
[2]College of Material and Textile Engineering, Jiaxing University, No.118 Jiahang Road. Jiaxing, 314000, P.R. China

Corresponding Author's Email: shengxie16@163.com

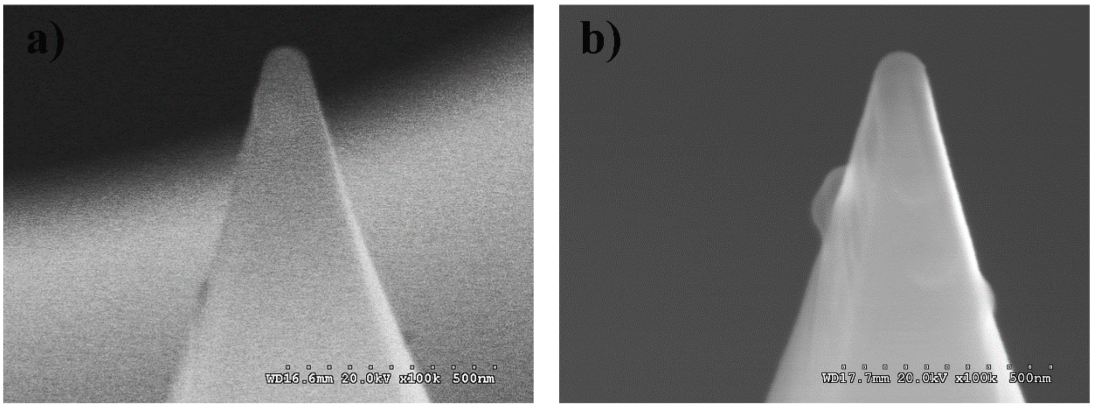

Figure S1. SEM images of the AFM tip a) before and b) after nanoindentation experiment.

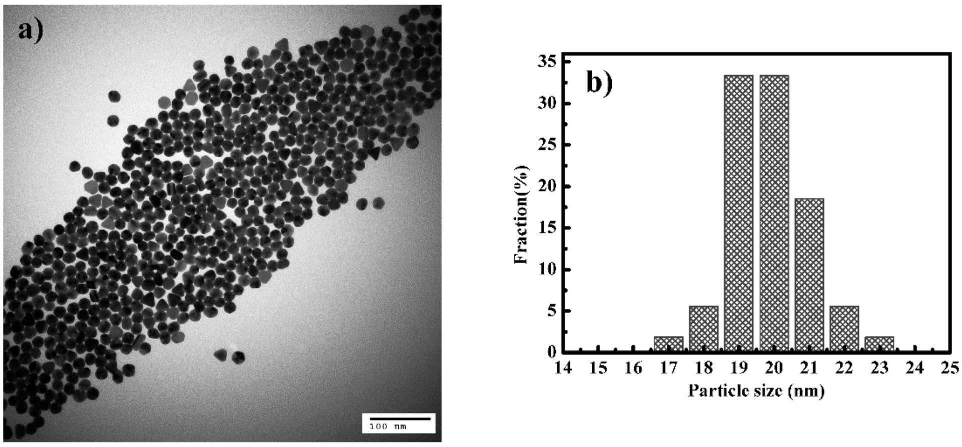

Figure S2.a) TEM image and b) particle size distribution of the gold nanoparticle.
